# Determination of HPV prevalence in oral/oropharyngeal mucosa samples in a rural district of São Paulo

**DOI:** 10.5935/1808-8694.20130107

**Published:** 2015-10-08

**Authors:** Vitor Breseghello Cavenaghi, Elias Jean Eid Ghosn, Natália Cruz, Lia Mara Rossi, Leonardo da Silva, Henrique Olival Costa, Luísa Lina Villa

**Affiliations:** a Medical Student- School of Medical Sciences - Santa Casa de São Paulo.; b Higher Education - Collaborator at the School of Medical Sciences - Santa Casa de São Paulo.; c PhD; Collaborator - School of Medical Sciences - Santa Casa de São Paulo.; d PhD; Assistant Professor of Otolaryngology - School of Medical Sciences - Santa Casa de São Paulo.; e PhD; Coordinator of the HPV Institute (INCT-HPV) - Santa Casa de São Paulo. School of Medical Sciences - Santa Casa de São Paulo.

**Keywords:** mouth, oropharynx, papillomavirus infections, polymerase chain reaction

## Abstract

Knowledge about HPV infection in the oral cavity/oropharynx may contribute to the elucidation of the role it plays in Head and Neck Squamous Cell Carcinoma (HNSCC).

**Objective:**

To determine the effectiveness of the methodology used for sampling the oral cavity and oropharynx mucosae and to determine the prevalence of HPV in the oral cavity and oropharynx of adults and children.

**Method:**

The study population was served by an assistance program in a rural district of São Paulo. The subjects were asked to donate samples regardless of complaints.

**Results and Conclusion:**

The study included 47 men, 77 women and 22 children, of which the oral cavity samples were obtained by gargling with commercially-available antiseptic mouthwash. We found 3 positive samples (2.4%) in adults: 2 HPV 55 and one HPV 58. No positive results were found in children. Furthermore we concluded that the sampling method with the mouthwash proved effective and fast for the detection of HPV in the oral cavity and oropharynx in the general population.

## INTRODUCTION

Human papillomavirus (HPV) is one of the most common causes of sexually transmitted infections worldwide. Although the causal relationship between high-risk HPV and cervical cancer is well established^1^, the implication of this viral infection in squamous cell carcinoma of the head and neck (HNSCC) remains uncertain. Brazil has one of the highest incidences of HNSCC on the planet, and 20,280 new cases were estimated for 2012^2^.

Much of this lack of knowledge happens because there is no conclusive evidence on the prevalence of oral cavity infection by HPV. Existing studies resort to various methods, either in sampling or in the detection of HPV, eventually producing different results.

Based on the literature, high-risk HPV types have been regularly detected in 16% to 33% in oral cavity squamous cell carcinoma (OCSCC), 28.2% to 47% in SCC of the oropharynx and 13.8% to 48.5% in laryngeal SCC^3^.

As observed in carcinomas of the cervix, HPV + OCSCCs are acquired predominantly via sexual transmission^4^, ^5^. However, especially in patients with laryngeal papillomatosis, vertical transmission from mother to fetus during pregnancy and childbirth may exist, indicating that HPV can be transmitted both sexually and non-sexually. HPV DNA detection in amniotic fluid, fetal membrane, umbilical cord and placental trophoblast cells suggest HPV infection in the uterus, or pre-natal^6^ transmission.

Since we have the availability of prevention tools such as the HPVvaccine^7^, and considering the logic that healthcare policies should determine in advance the impact of any intervention according to their cost/benefit analysis, we observed that there is a need to establish regional parameters in order to choose populations which are more adequate for OCSCC prevention. Knowing the continental dimensions of Brazil, the use of a simple, fast and effective way to check for HPV prevalence, it may help to lead more studies in different regions of the country and the world.

The objectives of this study were to investigate the usefulness of employing oral antiseptic mouth rinse in order to collect samples from the oral cavity and oropharynx, comparing the prevalence of HPV in the sample with that in the literature.

## METHOD

This study was carried out with prior approval from the Ethics Committee on Research with protocol number: 419/10.

The population under study participated in the assistance program conducted in 2011 in Votuporanga (latitude 20° 25'22 “South and longitude 49° 58'22” West) in São Paulo, Brazil. The entire population served by the assistance program was submitted to an ENT examination, regardless of complaints during the interview and screening. All patients participating in the assistance program conducted in the city, who were informed about the research and agreed to participate after signing the consent form, were enrolled in the program. We took off the study those who refused to participate in it.

Our study had men, women and children from 4 years of age. We collected samples from the mouth and oropharynx cells of the patients by peeling the skin after rinsing and gargling with commercial oral antiseptic (Alcohol-free Plax and Colgate). The subjects were instructed to avoid consumption of any food or liquid and remove dentures about 30 minutes before collection. The participant was then instructed to rinse the oral cavity with a single dose of 15 ml of mouthwash, gargling vigorously for about 15 seconds involving the entire surface of the mouth. Then, the subject was told to tilt the head back and gargle for another 15 seconds. After this time, the solution was spat back to a previously labeled 50 ml conical tube.

Immediately after collection, all samples were kept in a refrigerator at 4°C for a maximum period of 48 hours, when they were transported under refrigeration to the city of São Paulo and processed. PCR and DNA extraction tests were performed according to routine protocol. The DNA was extracted and purified according to instructions from Roche's AmpliLute Liquid Media Extraction Kit (Roche Diagnostics Mol, California, USA), and quantified in a full spectrum Nanodrop spectrophotometer. Equal amounts of DNA were subjected to HPV DNA detection and typing by polymerase chain reaction (PCR) using the Linear Array HPV Genotyping kit (Roche Mol Diagnostics, CA, USA). Biotinylated primers were used for a polymorphic region of 450 L1 HPV genome base pairs and another couple of primers for the human beta-globin gene used as DNA quality control, following manufacturer's instructions. This method enables the detection of 37 different types of HPV and has been validated in several studies carried out worldwide.

## RESULTS

Of the 166 individuals invited to the study, 145 (87.3%) agreed to participate, signed a consent form and were included in the study. Samples were collected from 77 adult women between 20 and 74 years of age (mean age ± SD = 45.3 ± 15.4), 47 adult males between 18 and 89 years of age (mean age ± standard deviation 51.4 ± 16.8) and 21 children between 4 and 17 years of age (mean age ± standard deviation 10.1 ± 4.2). Table 1 shows the sociodemographical characteristics of the population participating in the study.

**Table 1:** Sociodemographical characteristics.

	Adults n (%)	Children n (%)
Participants	124	21
Mean age (in years)	47.61	10.1

Gender		

Males	47 (37.9)	7 (33.3)
Females	77 (62.1)	14 (66.7)

Schooling		

Illiterate	19 (15.4)	3 (14.3)
Basic Education	75 (60.5)	13 (61.9)
High School	23 (18.5)	5 (13.8)
Higher Education	7 (5.6)	0 (0)

Race		

White	84 (67.7)	12 (57.1)
Black	13 (10.4)	4 (19)
Brown	27 (21.9)	4 (19)
Yellow	0 (0)	1 (4.9)

Marital Status		

Single	22 (17.7)	20 (95.2)
Married	82 (66.1)	1 (4.8)
Divorcee	8 (6.4)	0 (0)
Widow(er)	12 (9.8)	0 (0)

Smoking		

Yes	93 (75)	0 (0)
No	31 (31)	21 (100%)

Alcoholic beverage		

Yes	46 (37.1)	2 (9.5)
No	78 (62.9)	19 (90.5)

The DNA was successfully isolated from all samples. In addition, all samples had beta-globulin, indicating good quality of the material submitted to PCR. Three positive results were obtained in adults, with a prevalence of 2.4%. The HPV types found were HPV 55 - in two samples and HPV 58 in one. No positive results were found in children (Table 2).

**Table 2:** HPV DNA prevalence in oral mucosa samples.

HPV	Number found n (%)	Types
Adults	124	-

Positive	3 (2.4)	2 positive for type 55 1 positive for type 58
Negative	121 (97.6)	-

Children	21	-

Positive	0 (0)	-
Negative	21 (100)	-

## DISCUSSION

In a study which assessed the prevalence of HPV infection in the oral and oropharyngeal mucosae from 1,688 healthy men from Mexico, USA and Brazil, the authors found 4% of HPV infections, and 1.3% were positive for HPV-16 - the most prevalent type in this population^8^. Considering only the adult population assessed in Votuporanga, the HPV prevalence was 2.4%. In the juvenile population, there was no HPV, an expected result, since studies show that the prevalence of HPV in the oral cavity of individuals under 18 is low - a factor that may be related to increased prevalence in subjects who have already started their sexual life^9^. Accordingly, in a study with 4,150 Polish children and adolescents between 10 and 18 years, the prevalence was 1.08%, and HPV type 11 was the most prevalent^10^.

In a study carried out in Esquenazi et al.^11^ the authors evaluated 100 healthy subjects using the mouth brush method and found 0% prevalence of HPV. Similarly, in a study of 30 women who had genital warts by the swab method - positive samples were also not found^12^. In another study with the swab method, the authors found 23.2% of positive samples for HPV in a population of 125 individuals without overt oral lesions^13^.

Kreimer et al.^8^ used the same protocol for the collection of cell samples from the oral cavity and the oropharynx adopted in this study. In the study by Durzyńska et al.^10^, the method used was rinsing with 5 ml of saline. Other studies used the swab method for obtaining samples^11^, ^12^, ^13^.

Of the three positive samples in the study, two of them were type 55 and one was HPV 58, coinciding with the types found in the papers by Kreimer et al.^8^, in adult men, and Durzyńska et al.^10^ in children.

The main limitations of this study are related to the low number of participants, the proportion of adults and children and the inability to characterize the population on habits and sexual behavior. However, the methodology demonstrates the feasibility of conducting a study on the prevalence of HPV in a region with low technological resources, limited time and space, with results similar to those available in the literature.

## CONCLUSION

The collection method using rinsing with mouthwash was fast and effective for the detection of HPV in the oral cavity and oropharynx evidenced by the presence of DNA in all the samples and by PCR control by the beta-globulin gene - positive in all samples.

We found three positive (2.4%) samples for HPV in the oral cavity and oropharynx of the adult population participating in the study and no positive sample among the children. The HPV types found were the HPV 55 in two specimens, and one with HPV 58. This data is in agreement with the literature, showing low HPV prevalence in the mouth and oropharynx of healthy individuals.
